# Supplementary material for: Assessment of neurovascular coupling and cortical spreading depression in mixed mouse models of atherosclerosis and Alzheimer’s disease
Source: eLife. 2022 Jan 11;11:e68242. doi: 10.7554/eLife.68242 (PMC8752088; doi:10.7554/eLife.68242)
Supplement: Transparent reporting form [file elife-68242-transrepform1.docx]

***eLife’s* transparent reporting form**

We encourage authors to provide detailed information *within their submission* to facilitate the interpretation and replication of experiments. Authors can upload supporting documentation to indicate the use of appropriate reporting guidelines for health-related research (see [EQUATOR Network](http://www.equator-network.org/%20)), life science research (see the [BioSharing Information Resource](https://biosharing.org/)), or the [ARRIVE guidelines](http://www.plosbiology.org/article/info:doi/10.1371/journal.pbio.1000412) for reporting work involving animal research. Where applicable, authors should refer to any relevant reporting standards documents in this form.

If you have any questions, please consult our Journal Policies and/or contact us: [editorial@elifesciences.org](mailto:editorial@elifesciences.org).

**Sample-size estimation**

- You should state whether an appropriate sample size was computed when the study was being designed
- You should state the statistical method of sample size computation and any required assumptions
- If no explicit power analysis was used, you should describe how you decided what sample (replicate) size (number) to use

Please outline where this information can be found within the submission (e.g., sections or figure legends), or explain why this information doesn’t apply to your submission:

The number of animals uses in the study can be found at the start of the methods section. No explicit power analysis was used to estimate sample size, however average numbers per group were based on our previous publications (Sharp et al, 2019 JCBFM & Shabir et al, 2020 Sci Rep; see references).

**Replicates**

- You should report how often each experiment was performed
- You should include a definition of biological versus technical replication
- The data obtained should be provided and sufficient information should be provided to indicate the number of independent biological and/or technical replicates
- If you encountered any outliers, you should describe how these were handled
- Criteria for exclusion/inclusion of data should be clearly stated
- High-throughput sequence data should be uploaded before submission, with a private link for reviewers provided (these are available from both GEO and ArrayExpress)

Please outline where this information can be found within the submission (e.g., sections or figure legends), or explain why this information doesn’t apply to your submission:

Each animal (each n number in each group which is the biological n) had surgery performed on them (thinned cranial window) and 2x session replicates were performed on each animal; 1 chronic and 1 acute imaging session (session replicate of 2 per biological n). Histological and genetic studies were performed on the same animals with isolated brain tissue. This protocol is discussed in detail within the methods section.

Technical repeats of each stimulation were performed; 30 consecutive trials (n=30 per 2s experiment) for each 2s stimulation experiment, and 15 consecutive trials for each 16s stimulation experiment. Furthermore, 2 lots of 2s and 16s experiments were performed under 100% oxygen and 21% oxygen thus each chronic and acute imaging session had 4 experiments and repeated measures obtained across all of these for HbT, HbR & HbO as well as MUA on acute imaging sessions.

**Statistical reporting**

- Statistical analysis methods should be described and justified
- Raw data should be presented in figures whenever informative to do so (typically when N per group is less than 10)
- For each experiment, you should identify the statistical tests used, exact values of N, definitions of center, methods of multiple test correction, and dispersion and precision measures (e.g., mean, median, SD, SEM, confidence intervals; and, for the major substantive results, a measure of effect size (e.g., Pearson's r, Cohen's d)
- Report exact p-values wherever possible alongside the summary statistics and 95% confidence intervals. These should be reported for all key questions and not only when the p-value is less than 0.05.

Please outline where this information can be found within the submission (e.g., sections or figure legends), or explain why this information doesn’t apply to your submission:

All statistical analyses that were performed are discussed in detail within the statistical analysis section of the methods. Furthermore, all p values, statistical tests and post-hoc multiple comparison tests are mentioned within the figure legends, along with n, in addition to error bars being SEM.

(For large datasets, or papers with a very large number of statistical tests, you may upload a single table file with tests, Ns, etc., with reference to sections in the manuscript.)

**Group allocation**

- Indicate how samples were allocated into experimental groups (in the case of clinical studies, please specify allocation to treatment method); if randomization was used, please also state if restricted randomization was applied
- Indicate if masking was used during group allocation, data collection and/or data analysis

Please outline where this information can be found within the submission (e.g., sections or figure legends), or explain why this information doesn’t apply to your submission:

With respect to atherosclerotic and mix-model mice, a random selection of WT or J20 animals were selected to be given rAAV8-mPCSK9-D377Y injection and be put on a Western diet. The remaining animals were used as WT controls or J20-AD animals.

**Additional data files (“source data”)**

- We encourage you to upload relevant additional data files, such as numerical data that are represented as a graph in a figure, or as a summary table
- Where provided, these should be in the most useful format, and they can be uploaded as “Source data” files linked to a main figure or table
- Include model definition files including the full list of parameters used
- Include code used for data analysis (e.g., R, MatLab)
- Avoid stating that data files are “available upon request”

Please indicate the figures or tables for which source data files have been provided:

MATLAB code used to produce each of the neurovascular figures have been provided with the data files. The raw haemodynamic and neural data in an Excel spreadsheet have also been provided from which statistical analyses and data visualisation was performed.
